# Supplementary figures and images for: Rapid and sensitive detection of gram-negative bacteria using surface-immobilized polymyxin B
Source: PLoS One. 2023 Aug 28;18(8):e0290579. doi: 10.1371/journal.pone.0290579 (PMC10461818; doi:10.1371/journal.pone.0290579)

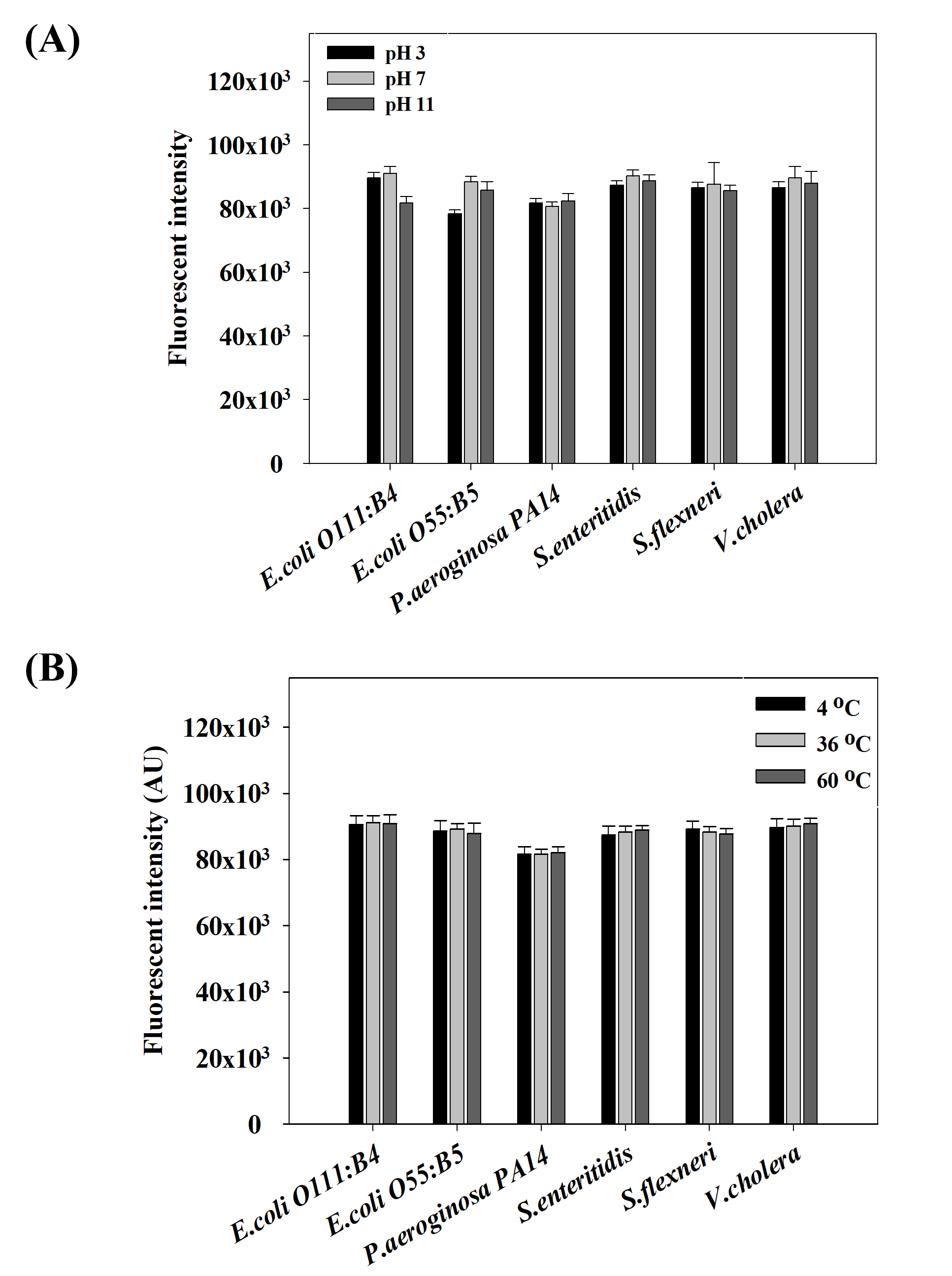

Supplement: S1 Fig — Sensitivity of the proposed PMB method under different (A) pH and (B) temperature conditions. The error bars indicate the standard deviation of five measurements. (TIF) [file pone.0290579.s001.tif]
