# Supplementary material for: Rapid and sensitive detection of gram-negative bacteria using surface-immobilized polymyxin B
Source: PLoS One. 2023 Aug 28;18(8):e0290579. doi: 10.1371/journal.pone.0290579 (PMC10461818; doi:10.1371/journal.pone.0290579)
Supplement: Attachment — Submitted filename: PONE-D-23-10755-Review comments.docx [file pone.0290579.s002.docx]

**Manuscript ID PONE-D-23-10755 : Major Revision**

This work described a method detecting gram-negative bacteria (GNB) based on specific binding of polymyxin B (PMB) to lipopolysaccharides (LPS) in outer membrane of GNB. GNB were initially bound to surface immobilized PMB, and FITC-conjugated PMB was then bound to the bound GNB for the detection by emiting fluorescence. The presence morphology of bacteria includes biofilm bacteria and planktonic bacteria. This method can count bacterioplanktonis under limited conditions and lack of experiments for counting biofilm bacteria. In addition, I wonder, it is unreasonable to counting dead cells by specific binding of PMB to LPS. Since most of the dead bacteria ruptured and split a large number of LPS into the medium, In that case，this method is difficult to count the samples containing both live bacteria and dead bacteria accurately by specific binding of PMB to LPS in the medium.Therefore, I wonder, how the authors prove that the method could count the number of dead bacteria or live bacteria in the clinical samples accurately.

The methods relied on the binding ability of LPS to PMB. When using PBS as a medium, the effect of different temperatures and PH on the accuracy of the method should be evaluated. This can reflect the stability of the method. In my opinion, the authors' method cannot accurately count specimens with bacterial death and LPS leakage and there will be a large error in biofilm bacteria. The authors found that the method was inaccurate in porcine plasma，but did not perform experiment such as PH or temperature.The influencing factors affecting the binding ability of polymyxin B and LPS should be evaluated which are the key points to the scope of application of this method. I recommend that authors deeply revise it and then resubmit it in a new face. There are more comments.

**Specific comments:**

1. Line 84-87: The source and name of the strains are not clear.
2. Line 263-264: The author said:“ the proposed method can detect dead GNB as well as live GNB”.I think the sentence lack of sufficient evidence. Most of the dead bacteria ruptured and split a large number of LPS into the medium. It is important to clarify the counting effect of this method on dead bacteria in such condition.
3. this method is difficult to count the samples containing both live bacteria and dead bacteria accurately by specific binding of PMB to LPS in the medium.
4. In Fig2, the authors did not show the length of the scale.
5. Line 294-295: It is suggested that the authors explore the mechanism of the components in porcine plasma affected the binding of GNB cells on the immobilized PMB and/or FITC conjugated PMB to GNB cells.
6. PH is commonly used to test the binding stability. The authors are advised to supplement this experiment.
