# Supplementary material for: Rapid and sensitive detection of gram-negative bacteria using surface-immobilized polymyxin B
Source: PLoS One. 2023 Aug 28;18(8):e0290579. doi: 10.1371/journal.pone.0290579 (PMC10461818; doi:10.1371/journal.pone.0290579)
Supplement: Attachment — Submitted filename: PONE-D-23-10755_Response letter.docx [file pone.0290579.s003.docx]

[Note: HTML markup is below. Please do not edit.]

Reviewers' comments:

Reviewer's Responses to Questions

**Comments to the Author**

1. Is the manuscript technically sound, and do the data support the conclusions?

The manuscript must describe a technically sound piece of scientific research with data that supports the conclusions. Experiments must have been conducted rigorously, with appropriate controls, replication, and sample sizes. The conclusions must be drawn appropriately based on the data presented.

Reviewer #1: Yes

Reviewer #2: Yes

2. Has the statistical analysis been performed appropriately and rigorously?

Reviewer #1: Yes

Reviewer #2: Yes

3. Have the authors made all data underlying the findings in their manuscript fully available?

The [PLOS Data policy](http://www.plosone.org/static/policies.action#sharing) requires authors to make all data underlying the findings described in their manuscript fully available without restriction, with rare exception (please refer to the Data Availability Statement in the manuscript PDF file). The data should be provided as part of the manuscript or its supporting information, or deposited to a public repository. For example, in addition to summary statistics, the data points behind means, medians and variance measures should be available. If there are restrictions on publicly sharing data—e.g. participant privacy or use of data from a third party—those must be specified.

Reviewer #1: Yes

Reviewer #2: Yes

4. Is the manuscript presented in an intelligible fashion and written in standard English?

PLOS ONE does not copyedit accepted manuscripts, so the language in submitted articles must be clear, correct, and unambiguous. Any typographical or grammatical errors should be corrected at revision, so please note any specific errors here.

Reviewer #1: Yes

Reviewer #2: Yes

5. Review Comments to the Author

Please use the space provided to explain your answers to the questions above. You may also include additional comments for the author, including concerns about dual publication, research ethics, or publication ethics. (Please upload your review as an attachment if it exceeds 20,000 characters)

**Reviewer 1**

**Manuscript ID PONE-D-23-10755: Major Revision**

This work described a method detecting gram-negative bacteria (GNB) based on specific binding of polymyxin B (PMB) to lipopolysaccharides (LPS) in outer membrane of GNB. GNB were initially bound to surface immobilized PMB, and FITC-conjugated PMB was then bound to the bound GNB for the detection by emiting fluorescence.

**C1.** The presence morphology of bacteria includes biofilm bacteria and planktonic bacteria. This method can count bacterioplankton is under limited conditions and lack of experiments for counting biofilm bacteria.

**Response:** The authors appreciate for the reviewer’s comment. In this study, we did not investigate sensitivity of the developed method for the biofilms. Instead, we tested the developed method for activated sludge floc consisted of microbial cells and their excreted materials called extra-polymeric substances (EPS), similar to biofilms. The counting sensitivity of the floc was slightly lower than that of the dispersed cells (86% of the dispersed cells), presumably due to difficulty of diffusion of the FITC-conjugated PMB to some microorganisms deeply localized within the floc (See Fig. 5 in the submitted manuscript). In response to the reviewer’s comment, we have not revised manuscript.

Fig. 5. Evaluation of cell aggregation for detecting GNB in the CFU assay and the proposed method. Activated sludge floc was used a form of aggregated cells. The error bars indicate the standard deviation of five measurements.

**C2.** In addition, I wonder, it is unreasonable to counting dead cells by specific binding of PMB to LPS. Since most of the dead bacteria ruptured and split a large number of LPS into the medium, In that case，this method is difficult to count the samples containing both live bacteria and dead bacteria accurately by specific binding of PMB to LPS in the medium. Therefore, I wonder, how the authors prove that the method could count the number of dead bacteria or live bacteria in the clinical samples accurately.

**Response:** The authors thank for the reviewer’s comment. We agree with the reviewer’s speculation that the some dead GNB ruptured and split a large number of LPS into the medium. Nevertheless, we found that the dead GNB possessed enough amount of LPS to be detectable by the PMB method developed in this study. Several studies also reported that some killed GNB still possess LPS (Ringdén, et al., 1977; van Langevelde, et al., 1998). However, the counting sensitivity of the dead cells represented by fluorescence intensity was slightly lower than that of live cells (91% of the live cells), presumably due to the reason speculated by the reviewer. In response to the reviewer’s comment, we have addressed the lower sensitivity for the dead cells, as follows.

**Page 13, line 271-273:**

“The counting sensitivity of the dead cells represented by fluorescence intensity was slightly lower than that of live cells (91% of the live cells), presumably due to some dead *E. coli* cells ruptured and split LPS into the medium.”

Fig. 6. Evaluation of viability of for detecting GNB in the CFU assay and the proposed method. *E. coli* cells were killed using 0.5% sodium hypochlorite for a preparation of dead GNB cells. The error bars indicate the standard deviation of five measurements.

**Reference**

Ringdén, O., Rynnel‐Dagöö, B., Waterfield, E. M., Möller, E., & Möller, G. (1977). Polyclonal antibody secretion in human lymphocytes induced by killed staphylococcal bacteria and by lipopolysaccharide. *Scandinavian Journal of Immunology*, *6*(11), 1159-1169.

van Langevelde, P., Kwappenberg, K. M., Groeneveld, P. H., Mattie, H., & van Dissel, J. T. (1998). Antibiotic-induced lipopolysaccharide (LPS) release from Salmonella typhi: delay between killing by ceftazidime and imipenem and release of LPS. *Antimicrobial agents and chemotherapy*, *42*(4), 739-743.

**C3.** The methods relied on the binding ability of LPS to PMB. When using PBS as a medium, the effect of different temperatures and PH on the accuracy of the method should be evaluated. This can reflect the stability of the method. In my opinion, the authors' method cannot accurately count specimens with bacterial death and LPS leakage and there will be a large error in biofilm bacteria. The authors found that the method was inaccurate in porcine plasma，but did not perform experiment such as PH or temperature. The influencing factors affecting the binding ability of polymyxin B and LPS should be evaluated which are the key points to the scope of application of this method. I recommend that authors deeply revise it and then resubmit it in a new face. There are more comments.

**Response:** The authors appreciate for the reviewer’s important comment and agree with the opinion. During optimization of the PMB-based method, we already tested the binding sensitivity between PMB and LPS under different pH and temperature conditions to optimize this newly developed method, but we did not include the results in the submitted manuscript. As shown in the figure below, sensitivities of the PMB method were not significantly affected by different pH and temperature for various GNB. In response to the reviewer’s comment, we have addressed the effects of temperature and pH in the revised manuscript. In addition, we have added the figures in the supplementary materials.

**Page 11, line 228-231:**

“pH and temperature may affect the sensitivity of the proposed PMB method, but experiments conducted for different pH (3, 7, 11) and temperature (4, 36, 60^o^C) for various GNB demonstrated insensitivity of the PMB method (Supplementary Fig. 1).”

**Supplementary Fig. 1**. Sensitivity of the proposed PMB method under different (A) pH and (B) temperature conditions. The error bars indicate the standard deviation of five measurements.

**Specific comments:**

**Q1.** Line 84-87: The source and name of the strains are not clear.

**Response:** The authors thank for the reviewer’s careful comment. In response to the reviewer’s comment, we have added the source and name of bacterial strains used in this study in the revised manuscript.

**Page 5, line 84-88:**

“Five species of GNB (*Escherichia coli* strain O111:B4, *Pseudomonas aeruginosa* PA14, *Sphingomonas paucimobilis* ATCC51231, *Pseudomonas alcaliphila* AL15-21T (JCM 10630T), and *Enterobacter cloacae* ATCC13047) and four species of GPB (*Staphylococcus aureus* ATCC6538, *Bacillus amyloliquefaciens* strain FXH73, *Bacillus thuringiensis* ATCC10792, and *Corynebacterium xerosis* ATCC7711) were used in this study.”

Fig. 2. Verification of the proposed method detecting GNB. a) CLSM images of DAPI (left), FITC (middle), and overlay of the two images (right) for the E. coli cells processed by the proposed method and stained with DAPI. Scale bars indicate 30 μm. b) FITC signal intensity for various GPB and GNB processed by the proposed method. Control indicates a sample without bacterial cells. The error bars indicate the standard deviation of five measurements. *, P > 0.10 versus control.

**Q2.** Line 263-264: The author said: “the proposed method can detect dead GNB as well as live GNB”. I think the sentence lack of sufficient evidence. Most of the dead bacteria ruptured and split a large number of LPS into the medium. It is important to clarify the counting effect of this method on dead bacteria in such condition.

**Response:** The authors thank for the reviewer’s comment. We agree with the reviewer’s speculation that the some dead GNB ruptured and split a large number of LPS into the medium. Nevertheless, we found that the dead GNB possessed enough amount of LPS to be detectable by the PMB method developed in this study. Several studies also reported that some killed GNB still possess LPS (Ringdén, et al., 1977; van Langevelde, et al., 1998). However, the counting sensitivity of the dead cells represented by fluorescence intensity was slightly lower than that of live cells (91% of the live cells), presumably due to the reason speculated by the reviewer. In response to the reviewer’s comment, we have addressed the lower sensitivity for the dead cells, as follows.

**Page 13, line 271-273**

“The counting sensitivity of the dead cells represented by fluorescence intensity was slightly lower than that of live cells (91% of the live cells), presumably due to some dead *E. coli* cells ruptured and split LPS into the medium.”

Fig. 6. Evaluation of viability of for detecting GNB in the CFU assay and the proposed method. *E. coli* cells were killed using 0.5% sodium hypochlorite for a preparation of dead GNB cells. The error bars indicate the standard deviation of five measurements.

**Reference**

Ringdén, O., Rynnel‐Dagöö, B., Waterfield, E. M., Möller, E., & Möller, G. (1977). Polyclonal antibody secretion in human lymphocytes induced by killed staphylococcal bacteria and by lipopolysaccharide. *Scandinavian Journal of Immunology*, *6*(11), 1159-1169.

van Langevelde, P., Kwappenberg, K. M., Groeneveld, P. H., Mattie, H., & van Dissel, J. T. (1998). Antibiotic-induced lipopolysaccharide (LPS) release from Salmonella typhi: delay between killing by ceftazidime and imipenem and release of LPS. *Antimicrobial agents and chemotherapy*, *42*(4), 739-743.

**Q3.** This method is difficult to count the samples containing both live bacteria and dead bacteria accurately by specific binding of PMB to LPS in the medium.

**Response:** The authors thank for the reviewer’s comment. We agree with the reviewer’s opinion that the PMB method cannot distinguish between live and dead GNB. As addressed in the submitted manuscript, we speculate that the feature can be a merit for testing clinical samples. LPS are major endotoxins that can elicit various immune responses via binding with toll-like receptors in human [31]. As such immune responses are initiated by LPS that are released by mostly dead GNB [8, 31], dead rather than live GNB pose more of a threat to human beings; thus, proper attention must be paid to dead GNB as well. In this respect, capability of dead GNB detection by the proposed method suggests it can be used for early detection of such risk elements. In response to this comment, we have not revised the manuscript.

**Reference**

[8] DeLucca AJ, Godshall MA, Palmgren MS. Gram-negative bacterial endotoxins in grain elevator dusts. American Industrial Hygiene Association Journal. 1984;45(5):336-9.

[31] Heumann D, Roger T. Initial responses to endotoxins and Gram-negative bacteria. Clinica Chimica Acta. 2002;323(1-2):59-72.

**Q4.** In Fig2, the authors did not show the length of the scale.

**Response:** The authors thank for the reviewer’s comment. In response to the reviewer’s comment, we have added the length of scale in the revised manuscript.

Fig. 2. Verification of the proposed method detecting GNB. a) CLSM images of DAPI (left), FITC (middle), and overlay of the two images (right) for the E. coli cells processed by the proposed method and stained with DAPI. Scale bars indicate 30 μm. b) FITC signal intensity for various GPB and GNB processed by the proposed method. Control indicates a sample without bacterial cells. The error bars indicate the standard deviation of five measurements. *, P > 0.10 versus control.

**Q5.** Line 294-295: It is suggested that the authors explore the mechanism of the components in porcine plasma affected the binding of GNB cells on the immobilized PMB and/or FITC conjugated PMB to GNB cells.

**Response:** The authors appreciate the reviewer’s question. Identification of the mechanism to lower the linearity in the test using porcine plasma compared with the test using PBS is beyond the scope of this research. However, we speculate that the component of porcine plasma such as proteins, nutrients, waste products, and electrolytes might affect the binding between PMB and LPS interaction. In response to the reviewer’s comment, we have added a sentence in the revised manuscript, as follows.

**Page 14, line 300-301:**

“Porcine plasma is reported to contain various proteins, nutrients, and electrolytes [32].”

**Reference**

[32] Engelking, L. R. (2015). Chapter 4-Protein Structure. Textbook of Veterinary Physiological Chemistry, 3, 18-25.

**Q6.** pH is commonly used to test the binding stability. The authors are advised to supplement this experiment.

**Response:** The authors agree with the reviewer’s comment on the importance of pH in the binding stability. As mentioned earlier, we have added the experimental result on different pH condition, as follow.

**Page 11, line 228-231:**

“pH and temperature may affect the sensitivity of the proposed PMB method, but experiments conducted for different pH (3, 7, 11) and temperature (4, 36, 60^o^C) for various GNB demonstrated insensitivity of the PMB method (Supplementary Fig. 1).”

**Supplementary Fig. 1**. Sensitivity of the proposed PMB method under different (A) pH and (B) temperature conditions. The error bars indicate the standard deviation of five measurements.

**Reviewer 2**

In this manuscript by Park and colleagues, a microscopy-based method for sensitive detection of GNB potentially from patient samples was described. The authors provided good rationale for the experiments and the experiments appear robust and statistically sound.

I only had a couple of follow up questions that would further strengthen the manuscript:

Q1. Please specify which polymyxin was used - polymyxin B or E?

**Response:** The authors thank for the reviewer’s kind comment. We used polymyxin B in the whole experiment. In response to the reviewer's comment, we have specified the polymyxin as “polymyxin B” in the revised manuscript.

Q2. The validation studies utilized an FITC conjugated PMB. Is the conjugation at a specific aa or nonspecific? How variable are the results (RFU detection and sensitivity) using multiple lots of FITC-PMB?

**Response:** The authors thank for the reviewer’s comment. We have used FITC using the LYNX rapid fluorescein antibody conjugation kit (Bio-Rad, Hercules, CA, USA) for generating FITC-PMB conjugation. When the kit used, FITC was binding to the primary amines and thiols (e.g. thiomersal/thimerosal) of PMB during the procedures. We addressed the conjugation method in the Materials and Method section. Additionally, the variability of FITC conjugation efficiency was not observed using multiple lots of FITC-PMB in our experiments.

Q3. The authors should compare different starting densities of GNB vs GPB to ensure that there would not be RFU associated with high concentrations of GPB.

**Response:** The authors appreciate for the reviewer’s critical comment. Unfortunately, we did not conduct experiments for different ratio between GNB and GPB. One reason of not to conduct such experiments was that several researchers reported that PMB have no affinity with GPB possessing a thick peptidoglycan layer and the absence of the outer membrane (Yin et al., 2020; Evans et al., 1999). Another reason was that the signal intensities were very low and not significantly different from those of the control (i.e., no cell condition), even under very high cell concentration (10^7^ cells/mL) for the various GNB (see below Fig. 2 (B)).

**Reference**

Yin, J., Meng, Q., Cheng, D., Fu, J., Luo, Q., Liu, Y., & Yu, Z. (2020). Mechanisms of bactericidal action and resistance of polymyxins for Gram-positive bacteria. Applied microbiology and biotechnology, 104, 3771-3780.

Evans, M. E., Feola, D. J., & Rapp, R. P. (1999). Polymyxin B sulfate and colistin: old antibiotics for emerging multiresistant gram-negative bacteria. Annals of Pharmacotherapy, 33(9), 960-967.

Fig. 2. FITC signal intensity for various GPB and GNB processed by the proposed method. Control indicates a sample without bacterial cells. The error bars indicate the standard deviation of five measurements. *, P > 0.10 versus control.

Q4. The authors should provide a rationale for why porcine plasma was used. What about whole blood as a sample matrix? Can the GNB be detected in whole blood? If not, why not?

**Response:** The authors appreciate for the reviewer’s comment. Although we have not included the result in the submitted manuscript, we conducted detection sensitivity of the PMB method using whole blood. As shown a figure below, the result showed that sensitivity as well as linearity of the relationship between cell numbers and fluorescent intensity were lower than those with porcine plasma.
